# Connectivity between surface and deep waters determines prokaryotic diversity in the North Atlantic Deep Water

**DOI:** 10.1111/1462-2920.13237

**Published:** 2016-03-30

**Authors:** Alexander H. Frank, Juan A. L. Garcia, Gerhard J. Herndl, Thomas Reinthaler

**Affiliations:** ^1^ Department of Limnology and Bio‐OceanographyUniversity of ViennaViennaAustria; ^2^ Department of Biological OceanographyRoyal Netherlands Institute for Sea ResearchTexelThe Netherlands

## Abstract

To decipher the influence of depth stratification and surface provincialism on the dark ocean prokaryotic community composition, we sampled the major deep‐water masses in the eastern North Atlantic covering three biogeographic provinces. Their diversity was evaluated using ordination and canonical analysis of 454 pyrotag sequences. Variance partitioning suggested that 16% of the variation in the bacterial community composition was based on depth stratification while 9% of the variation was due to geographic location. General linear mixed effect models showed that the community of the subsurface waters was connected to the dark ocean prokaryotic communities in different biogeographic provinces. Cluster analysis indicated that some prokaryotic taxa are specific to distinct regions in bathypelagic water masses. Taken together, our data suggest that the dark ocean prokaryotic community composition of the eastern North Atlantic is primed by the formation and the horizontal transport of water masses.

## Introduction

The ocean harbors about 10^29^ prokaryotic cells with an enormous phylogenetic diversity (Whitman *et al*., 1998). In terms of volume, the open ocean below 200 m depth represents the largest biome on earth with Bacteria and Archaea playing a major role in the organic matter cycling in the meso‐ and bathypelagic waters (Azam *et al*., 1983; Reinthaler *et al*., 2010). However, our knowledge on microbial taxa and their traits in the dark ocean is still limiting our mechanistic understanding of the functioning of dark ocean microbial communities.

For the surface ocean, annually repeatable patterns in bacterial community composition have been found, associated with recurring physico‐chemical changes in the environment (Fuhrman *et al*., 2006; Vergin *et al*., 2013). The distribution of surface water bacterial communities also suggested a bipolar distribution where the community composition in the Arctic and Antarctic regions was more similar to each other than compared to those at lower latitudes (Ghiglione *et al*., 2012). Moreover, an extended global epipelagic dataset indicated biogeographic patterns of Bacteria similar to macroscopic organisms (Sul *et al*., 2013). The reason for the global distribution pattern of open ocean Bacteria is still controversial and might be the result of dispersal limitation or environmental selection (Martiny *et al*., 2006). A recent simulation using a global surface ocean circulation model supports the notion that neutral evolution in combination with dispersal limitation generates biogeographic patterns in bacterial community composition in surface waters (Hellweger *et al*., 2014).

In addition to the latitudinal trends, prokaryotic community composition in the ocean is depth‐stratified (DeLong *et al*., 2006; Brown *et al*., 2009), potentially linked to depth‐related changes in the quality and quantity of available energy sources. It is generally assumed that the pycnocline at the periphery of distinct deep‐water masses is a major constraint for the unrestricted, vertical distribution of the free‐living prokaryotes to the dark ocean. This dispersal limitation may result in an apparent depth stratification of the free‐living non‐sinking prokaryotic community (Galand *et al*., 2010; Agogué *et al*., 2011). In contrast, microbial communities attached to sinking particles are likely able to cross density gradients and thus might significantly contribute to the diversity of deep‐water prokaryotic communities (Nagata *et al*., 2010; Moeseneder *et al*., 2012), particularly if the sinking particles enter denser deep waters and thereby become neutrally buoyant (Bochdansky *et al*., 2010). The extent of surface derived prokaryotes shaping deep‐water diversity depends on the particle properties and thus indirectly on the surface water primary production (Cram *et al*., 2015), the strength of water column stratification and the source community composition (Hewson *et al*., 2006; Herndl and Reinthaler, 2013).

Oceanic regions with similar physical and chemical conditions and dominant plankton species have been assigned as distinct biogeographic provinces (Longhurst *et al*., 1995; Oliver and Irwin, 2008). The widely adopted scheme by Longhurst (2007) suggests several biogeographic provinces based on wind regimes, water column structure, nutrient concentrations, satellite‐derived primary production, and phytoplankton community composition. Although this classification is based on surface ocean properties, individual biogeographic provinces might be connected to a variable extent to the processes in the deeper water column.

Thus far, only few studies have examined prokaryotic activity and diversity in relation to biogeographic provinces in the ocean. Furthermore, coherent datasets considering biogeographic provinces and the structuring forces of the deep‐water masses on microbial communities are essentially missing. Studies conducted in the western North Atlantic suggest that bacterial abundance and prokaryotic heterotrophic production in the meso‐ and bathypelagic waters of the North Atlantic roughly reflect biogeographic provinces (De Corte *et al*., 2012).

**Figure 1:**
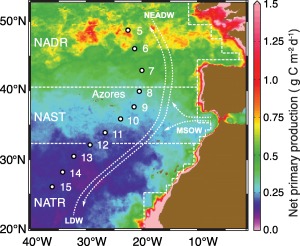
Map of occupied stations in the North Atlantic. The stations are indicated by white dots and numbers. Borders between biogeographic provinces are indicated by white dashed lines (NADR—North Atlantic Drift Province. NAST—North Atlantic Subtropical Province. NATR—North Atlantic Tropical Gyral Province). Net primary production (g C m^−2^ day^−1^) on the day of sampling is color‐coded. The flow of the main deep water masses is indicated by white lines and arrows. MSOW—Mediterranean Sea Outflow Water, NEADW—North East Atlantic Deep Water, LDW—Lower Deep Water.

In the current study, we explicitly addressed the question of the connectivity between the dark ocean prokaryotic community composition and the surface ocean biogeographic provinces. Samples were obtained along a 3000 km long transect in the eastern North Atlantic (Fig. 1). We estimated the prokaryotic community composition in the subsurface waters influenced by atmospheric conditions and in five major deep‐water masses over three biogeographic provinces. DNA was sequenced using pyrosequencing technology to include the rare biosphere (Sogin *et al*., 2006). Our analyses suggest that water mass association is the predominant predictor of prokaryotic community composition in the dark ocean. However, a considerable amount of variation in community changes is attributable to the biogeographic provinces of the North Atlantic surface waters.

## Results

### Sequencing effort and yield

The primer set yielded a total of 168,634 sequences with an average of 1,971 ± 665 reads and an average read length of 370 ± 44 bp per sample. Sequence analysis identified a total of 1,544 bacterial OTUs with an average of 157 ± 35 per sample.

### General depth distribution of OTUs

A general categorization of the OTU depth distribution indicated that ∼12% of all bacterial OTUs were present exclusively in the SSL, 26% were specific to the mesopelagic (O_2_‐min, MSOW, and AAIW) while the bathypelagic water masses (NEADW and LDW) harbored 19% unique OTUs; 23% were found in both, the meso‐ and bathypelagic layer (Supporting Information Fig. S1).

### Alpha—Diversity

**Figure 2:**
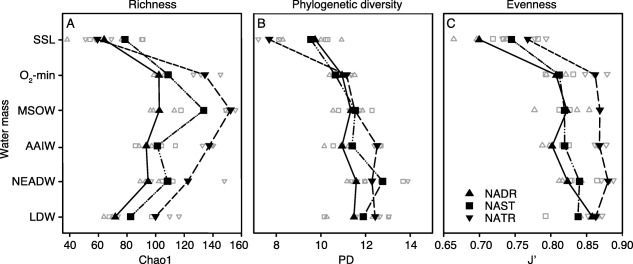
Rarefied *alpha*‐diversity of the water masses and provinces. Filled symbols depict the mean, open symbols indicate the results of individual samples. (A) Chao1 index, (B) phylogenetic diversity (PD), and (C) Pilou's evenness (*J*) in the different the water masses: SSL—subsurface layer (100 m depth), O_2_‐min—oxygen minimum, MSOW—Mediterranean sea outflow water. AAIW—Antarctic Intermediate Water: NEADW—North East Atlantic Deep Water, LDW—Lower Deep Water. The individual samples are indicated with the respective province: NADR—North Atlantic Drift Province. NAST—North Atlantic Subtropical Province. NATR—North Atlantic Tropical Gyral Province.

Sample coverage of the bacterioplankton was > 80% in all samples, as indicated by rarefaction curves (Supporting Information Fig. S2). The Chao1 index, used as index for OTU‐based *alpha*‐diversity, changed significantly between the water masses (ANOVA, *F*_5,44_ = 20.33, *p* < 0.001) and biogeographic provinces (ANOVA, *F*_2,47_ = 17.75, *p* < 0.001, Fig. 2A). On average, the MSOW, the AAIW and the NEADW exhibited a significantly higher diversity (Chao1 = 118 ± 8) as compared to the SSL, the O_2_‐min and the LDW (Chao1 = 87 ± 20). Additionally, *alpha*‐diversity increased from the North Atlantic Drift Province to the North Atlantic Gyral Province (Tukey HSD, *p* < 0.001; Fig. 2A, Supporting Information Table S1) while the influence of the water masses on diversity was independent from the biogeographic provinces.

The phylogenetic diversity (PD), however, increased from the SSL towards the NEADW (ANOVA, *F*_5,44_ = 13.99, *p* < 0.001, Fig. 2B). Using phylogenetic diversity, we found no significant influence of the biogeographic provinces on the phylogenetic diversity as determined by a two‐way ANOVA (Fig. 2B).

Pielou's evenness (*J*′) was significantly lower in the SSL compared to all the other water masses (ANOVA, *F*_5,44_ = 24.48, *p* < 0.001) and varied over a relatively narrow range from the mesopelagic to the bathypelagic water masses (Fig. 2C). Evenness of the North Atlantic Drift Province (NADR; 0.800 ± 0.006) and the North Atlantic Subtropical Province (NAST; 0.812 ± 0.007) were significantly lower than in the southern North Atlantic Tropical Gyral Province (0.851 ± 0.006; NATR; ANOVA, *F*_2,47_ = 12.71, *p* < 0.001, Fig. 2C).

### Beta—Diversity

**Figure 3:**
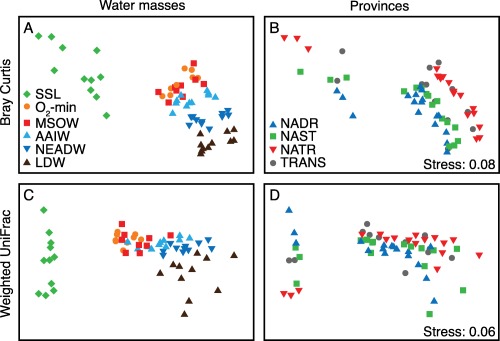
Nonmetric multidimensional scaling (NMDS) plots of community composition using Bray–Curtis similarity and bacterial OTUs (A, B) and weighted Unifrac dissimilarity (C, D). Samples are color coded according to water mass in (A) and (C) or biogeographic province in (B) and (D). SSL—subsurface layer (100 m depth), O_2min_—oxygen minimum, MSOW—Mediterranean Sea Outflow Water. AAIW—Antarctic Intermediate Water: NEADW—North East Atlantic Deep Water, LDW—Lower Deep Water. The individual samples are indicated with the respective province: NADR—North Atlantic Drift Province. NAST—North Atlantic Subtropical Province. NATR—North Atlantic Tropical Gyral Province. TRANS indicates samples lacking distinct affiliation to one province.

The communities clustered according to water masses as derived from the nonmetric multidimensional scaling (NMDS) plots based on Bray–Curtis similarity of the Hellinger‐transformed OTU table (Fig. 3A). This strong effect of water masses was also supported by a two‐way ANOSIM (global *R* = 0.85, *p* < 0.001). A similar clustering was obtained in plots based on weighted UniFrac (Fig. 3C).

A clear separation of the community according to biogeographic provinces was obtained between the northern provinces (NADR and NAST) and the most southern province (NATR) based on NMDS (Fig. 3B). However, less variability was explained as compared to the influence of water masses (two‐way ANOSIM, *R* = 0.059, *p* < 0.001). Using weighted UniFrac analysis, the clustering according to biogeographic provinces in the NMDS seemed complex but was statistically significant [ANOSIM (NADR, NATR), *R* = 0.70, *p* < 0.001; Fig. 3D]. This clustering remained significant even after removing the rather strong influence of the SSL [ANOSIM (NADR, NATR), *R* = 0.63, *p* < 0.001].

Additionally, the OTU based analysis suggested that bacterial community composition changed within water masses between biogeographic provinces, as indicated by the uniform distances from the northern provinces (NADR and NAST) to the North Atlantic Tropical Province (NATR) for each water mass (Fig. 3A,B). Moreover, the weighted UniFrac analysis suggested that the latitudinal influence on the bacterial community composition was dependent on the specific water mass. For example, changes between provinces were minor for the O_2_‐min layer and more pronounced in the LDW. Similar to the OTU based analysis, analysis of similarity on the weighted UniFrac analysis indicated close resemblance of the community composition of O_2_‐min layer and MSOW, but also of the MSOW and the AAIW. In contrast to the OTU‐based analysis, however, AAIW showed some similarity with the NEADW (ANOSIM, *R* = 0.38, *p* < 0.05), indicated by the overlapping clusters of both water masses (Fig. 3C).

**Figure 4:**
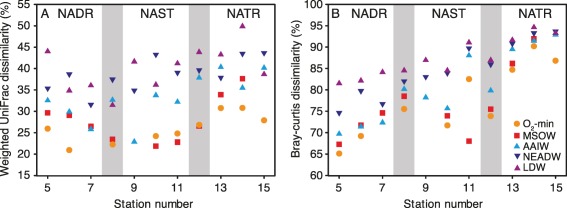
Dissimilarity of water‐mass specific communities to the epipelagic community composition given as weighted UniFrac (A) and the Bray‐Curtis dissimilarity metric (B) at each station from north (station 5) to south (station 15) crossing three Provinces: NADR—North Atlantic Drift Province, NAST—North Atlantic Subtropical Province, NATR—North Atlantic Tropical Gyral Province. Grey bars indicate transition zones between provinces. Samples are color coded according to water mass. O_2_‐min—oxygen minimum, MSOW—Mediterranean Sea Outflow Water, AAIW—Antarctic Intermediate Water, NEADW—North East Atlantic Deep Water, LDW—Lower Deep Water.

We performed linear mixed effect models (LME) on the weighted UniFrac distance matrix. The Linear mixed effect models indicated a significant province‐dependent change in the link between the bacterial community composition of the SSL and the underlying water masses (Fig. 4). The dissimilarity between the bacterial community composition of the SSL and the deep communities increased by ∼21% from the North Atlantic Drift Province to the North Atlantic Tropical Gyral Province (LME, chi‐square_10_ = 27.0, *p* < 0.01). This province‐dependent change of similarity between the bacterial community composition of the SSL and the deep‐water masses was least in the LDW (15%) and greatest in the AAIW (31%).

### Relative abundance of bacterioplankton phylotypes in the water masses

**Figure 5:**
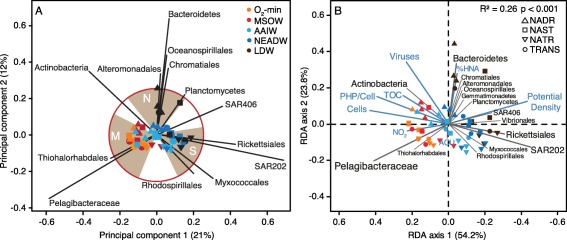
Principal component analysis (PCA) and redundancy analysis (RDA) of meso‐ and bathypelagic phylotypes. In the PCA (A) only phylotypes contributing to the spreading more than the average (indicated by the red circle) are indicated. Brown pie slices within the circle indicate northern (N) and southern (S) regions of the bathypelagic, as well as the mesopelagic realm (M). In RDA (B), the significant environmental response‐vectors are shown (downscaled by the factor 2). Vectors for species and constrained sites in the RDA are scaled to the square root of their Eigen value. Colors indicate water masses, symbol shapes correspond to provinces. The explained variation in percent is given for the two main axes, which are both highly significant. Abbreviation of the water masses and provinces as follows: SSL—subsurface layer (100 m depth), O_2_‐min—oxygen minimum, MSOW—Mediterranean Sea Outflow Water, AAIW—Antarctic Intermediate Water, NEADW—North East Atlantic Deep Water, LDW—Lower Deep Water. The individual samples are indicated with the respective province: NADR—North Atlantic Drift Province, NAST—North Atlantic Subtropical Province, NATR—North Atlantic Tropical Gyral Province.

The mean relative abundances of the dominant phylotypes in the water masses indicated a major contribution of *Pelagibacteraceae* (*Alphaproteobacteria*), decreasing from 61% in the SSL to 39% in the LDW (Supporting Information Fig. S3). An unclassified member of the *Deltaproteobacteria* related to the SAR324 cluster (Sva0853) contributed substantially to the deep‐water masses, i.e., in the range of 13–18%. The relative abundance of members of the SAR202 and SAR406 clusters increased with depth, contributing on average 8% to the bacterioplankton community in the NEADW and LDW (Supporting Information Fig. S3). Other groups such as the *Desulfobacterales*, *Thiohalorhabdales* (*Gammaproteobacteria*) and the *Planctomycetes* were present in all water masses, albeit at low relative abundances (Supporting Information Figs. S3 and S4). In contrast to the NADR, the community changes in the southern province NATR were largely influenced by several groups of low abundance bacterial taxa (SAR406 cluster, *Rickettsiales*, SAR202 and *Pseudoalteromonadales*) as indicated by the RDA triplot depicting the correlation of phylotypes with environmental factors (Fig. 5B).

### Distribution and variability of phylotypes

The distribution and relative dominance of phylotypes (as defined in the “Methods” section) across the water masses and biogeographic provinces were analyzed by principal component analysis (PCA). The SSL showed a distinctly different composition in bacterial phylotypes as compared to the deep‐water masses and was dominated by *Pelagibacteraceae, Bacteroidetes*, *Actinobacteria*, *Cyanobacteria*, *Rhodobacterales*, and *Oceanospirillaceae*. For evaluating the changes in the meso‐ and bathypelagic bacterial community composition, the data of the SSL were removed from the analysis to increase the analytical resolution of the vertical and horizontal changes in relative phylotype abundance in the meso‐ and bathypelagic water masses along the transect. Two main axes were retained in the principal component analysis and accounted for 42% of the variation in the dataset (Fig. 5A). A potential third principal component axis was driven by the relative abundance of *Vibrionales* (*Gammaproteobacteria*) that peaked in the LDW south of the Azores (Station 11) at a relative abundance of 29% and sharply decreased to the north and to the south. PCA indicated latitudinal and water mass‐related changes in bacterial community composition in the AAIW, NEADW, and LDW in addition to the varying dominance of specific phylotypes (Fig. 5A). Similar to weighted UniFrac, the latitudinal changes in the MSOW and O_2_‐min layer were less pronounced compared to the deeper water masses.

Overall, PCA indicated a decreasing relative abundance of *Pelagibacteraceae* with depth, representing a major contribution to the changing bacterial community composition from the meso‐ to the bathypelagic water masses (Fig. 5A). Similarly, the *Thiohalorhabdales* significantly deceased with depth, while the SAR406 cluster significantly increased with depth. In the NEADW and LDW of the North Atlantic Drift Province, mainly subgroups of *Bacteroidetes* (*Flavobacteria*), *Planctomycetes* (*Phycisphaera*), and *Gammaproteobacteria* (*Alteromonodales*, *Oceanospirillales*, *Chromatiales*) structured the bacterioplankton. Despite exhibiting a lower relative abundance than the former, some *Alphaproteobacteria* (i.e., *Rickettsiales* excluding SAR11) together with members of the SAR202 cluster were responsible for the variability in the bathypelagic community composition in the southern provinces NAST and NATR. *Actinobacteria* (*Acidimicrobia*) were relatively more important in the northern part of the transect while *Rhodospirillales*, and *Deltaproteobacteria* (*Myxococcales*) showed a relatively higher abundance toward the south. These groups substantially influenced the latitudinal changes of the bacterial community composition in the mesopelagic water masses (Fig. 5A).

### Environmental parameters influencing bacterial community composition

Changes in the bacterial community composition in relation to the environment were explored by redundancy analysis (RDA). Bacterial phylotypes in the deep‐water masses were positively correlated with several environmental and biotic parameters (Fig. 5B). RDA indicated a correlation of total organic carbon (TOC) with latitude despite its rather low variability along the transect (Supporting Information Table S2). Additionally, the increasing TOC concentrations towards the high latitudes correlated with the increasing abundance of *Actinobacteria* and *Thiohalorhabdales* within the mesopelagic waters (Fig. 5B). Particularly in the North Atlantic Drift Province, the percentage of high nucleic acid cells (HNA) increased with depth and correlated with *Alteromonodales*, *Bacteroidetes*, *Oceanospirillales*, and *Chromatiales* (compare Fig. 5A and B). *Actinobacteria*, dominating in the North Atlantic Drift Province, correlated with viral abundance that, in turn, generally decreased towards the NAST and NATR in the meso‐ and bathypelagic water masses. RDA also suggested a link between increasing cell‐specific heterotrophic production in the O_2_‐min and the MSOW and the relative abundance of *Thiohalorhabdales*.

**Figure 6:**
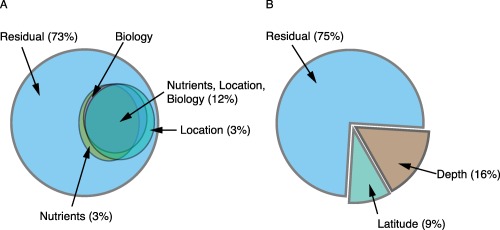
Venn diagram and pie chart of the variance partitioning analysis. A model of bacterial phylotypes including the complete set of environmental parameters (A) and a reduced model using depth and latitude (B) is shown. The areas correspond to the amount of variance explained by each factor. Overlapping areas indicate shared variation of the parameter effect on community composition in (A).

Variance partitioning of the RDA showed that 27% of the total variability in the community composition below the SSL were explained by the measured environmental variables (Fig. 6A). A rather small fraction of the variability (3% each) was exclusively associated with location (geographic position and depth) or chemical parameters (salinity, O_2_, AOU, TOC, PO_4_, SiO_4_, and NO_3_). Adding biological parameters (viral and prokaryotic abundance, percentage of HNA, ectoenzyme activity and cell‐specific leucine incorporation) did not increase the explained overall variance. A more reduced model using depth and latitude had a similar explanatory power (25%) as compared to the more complex model including all measured variables (Fig. 6B).

## Discussion

### Prokaryotic diversity in the deep North Atlantic

In the subsurface layer, an increase in OTU‐based bacterial *alpha*‐diversity was detected from the North Atlantic Drift Province to the North Atlantic Tropical Province (Fig. 2A). This was also reported in a global study on bacterial richness, where an increasing diversity towards lower latitudes has been found (Fuhrman *et al*., 2008). Also, a meta‐analysis showed significant latitudinal gradients in bacterial diversity, both in the southern and northern hemisphere (Sul *et al*., 2013).

The richness‐based *alpha*‐diversity indices have been questioned, however, because changes in the community composition cannot be derived from OTU abundances alone (Hanson *et al*., 2012). Thus, in an attempt to circumvent some of the ambiguities concerning current OTU‐based *alpha*‐diversity measures, we additionally analyzed the data using phylogenetic diversity (Faith, 1992). In contrast to OTU richness, this approach showed that diversity increased with depth and decreased with temperature (Fig. 2B) coinciding with a decreasing productivity over the broad scale of environmental parameters obtained from the lower epipelagic towards the LDW. It has been shown that evenness supports functional stability of ecosystems (Wittebolle *et al*., 2009). In our study evenness indicated a similar pattern in all three provinces with relatively low values in the SSL and higher evenness throughout much of the deep‐water column. A higher taxonomic evenness in the bathypelagic, with an overall higher variability was also reported in earlier studies (Hewson *et al*., 2006; Agogué *et al*., 2011).

*Beta*‐diversity is characterized by partitioning diversity among communities or along environmental gradients using the number of shared species between two communities (Lozupone and Knight, 2008). Mixed effect models on the dissimilarity between the epipelagic and the respective deep‐water communities suggested that the changes in the prokaryotic community composition of the deep‐water masses are connected to the latitudinal changes in the epipelagic community of the different biogeographic provinces. Furthermore, the relation between the epipelagic bacterial communities and the respective deep‐water mass associated community was dependent on the province, suggesting a variable degree of connectivity of surface water and dark ocean community composition (Figs. 3A,B and 4B). Prokaryotic communities might evolve with ageing of a particular water mass and the associated changes in the physicochemical characteristics. However, no such effect was found in this study. In contrast, a low but significant similarity was obtained between communities in the same water mass at different locations along the transect (Fig. 3). Thus, our data suggest that dispersal limitation is decoupling the communities of adjacent water masses, while the productivity of the surface community is modulating the intensity of the epipelagic influence on the deep‐water masses.

In contrast to methods that are purely based on species counts, weighted Unifrac seems robust to variations in the region sequenced (Liu *et al*., 2007) and also chimeras have been shown to not affect network clustering of Unifrac distances (Ley *et al*., 2008). The major pattern of an increasing north to south dissimilarity between the SSL communities and the bathypelagic water masses was also revealed analyzing the weighted Unifrac distance plots (Fig. 3C and D). We suggest that differences between weighted UniFrac and Bray–Curtis distances are due to changes in phylogenetic affiliation not resolved in the analysis using OTUs. It has been proposed that phylogenetic similarity and functional traits are connected to a certain degree (Philippot *et al*., 2010; Ortmann and Ortell, 2014) although the extent of the link between phylogeny and metabolic function is still under debate (Prosser *et al*., 2007). In our study, the weighted Unifrac plots indicated a high similarity of the AAIW and the MSOW/O_2_‐min layer in the North Atlantic Drift Province, while in the oligotrophic gyre (NATR) the AAIW was more similar to the NEADW. Similarly, the linear mixed effect model also suggested a higher connectivity of the AAIW to the SSL in the NADR as compared to the NATR. This might indicate relatively less dispersal of the surface bacterial community into the mesopelagic waters in the subtropical gyre than at higher latitudes where export flux of particles is higher and water column stratification less intense (Buesseler and Boyd, 2009). A recent study suggests that the particle‐association lifestyle is conserved within the phylogeny of the bathypelagic prokaryotes (Salazar *et al*., 2015). Therefore, the apparent change in connectivity between epipelagic and mesopelagic waters in the different provinces might be due to environmental filtering or directly related to province‐specific particle flux and its attached microbial community.

### Biogeography of abundance categories

Typically, natural prokaryotic communities are characterized by a large number of low abundance OTUs coined the ‘rare biosphere’ (*sensu* Sogin *et al*., 2006). This rare biosphere might constitute a seed bank and become abundant once environmental conditions change (Pedros‐Alio, 2006). Additionally, some members of the rare biosphere may influence biogeochemical cycles disproportionally to their (low) abundance (Fuhrman, 2009). To test the impact of the rare bacterial biosphere on the changes in diversity, we assigned the OTUs to three different groups according to their relative abundance. Only ten OTUs exceeded at least once a relative abundance of 10% of the per‐sample total OTU counts and thus were categorized as abundant. In total, 122 OTUs constituted between 1 and 10% of the bacterial community, categorized as common. Based on the sequencing effort (∼1,500 sequences per sample), we considered a relative abundance of <1% as rare OTUs. This low abundance fraction was by far the most diverse category comprising ∼1,410 bacterial OTUs (data not shown). The general resemblance pattern between deep‐water communities was similar for the three abundance categories (Mantel test based on weighted Spearman: *R* > 0.8, *p* < 0.001 for all three abundance categories combined; Supporting Information Table S3). This finding is in agreement with a report on Arctic bacterioplankton where the rare Bacteria exhibited a comparable biogeographic distribution pattern as the abundant taxa (Galand *et al*., 2009). However, low abundance taxa are apparently more important for the overall changes in community composition in deep‐water masses than the abundant taxa (Supporting Information Table S3 and Supporting Information Fig. S5), a finding also reached by Agogué *et al*. (2011).

### Distribution patterns of bacterial phylotypes

Similar to other reports from the region (Schattenhofer *et al*., 2009; Agogué *et al*., 2011), the ubiquitous SAR11 clade (*Pelagibacteraceae*) dominated the relative abundance of the prokaryotic community in the sub‐surface layer and decreased to the LDW (Supporting Information Fig. S3). The relative abundance of *Bacteroidetes* increased in the LDW flowing from the south to the north and correlated with the percentage of high nucleic acid (HNA) containing cells (Fig. 5B). *Bacteroidetes* are known to be associated with phytoplankton blooms and marine snow particles (DeLong *et al*., 1993; Rath *et al*., 1998; Fandino *et al*., 2001). Thus, it seems likely that in the productive northern North Atlantic *Bacteroidetes* is transported into the deep waters with senescent and sedimenting phytoplankton blooms and marine snow. Members of the *Oceanospirillales* were mainly encountered in the LDW of the North Atlantic Drift Province (Fig. 5 and Supporting Information Fig. S4). Recently piezophilic *Oceanospirillales* have been isolated, thriving at depth between 2000 and 7000 m (Cao *et al*., 2014), and some members of this group also contain RuBisCO and sulfur‐oxidation genes that may enable them to switch between an auto‐ and mixotrophic lifestyle (Swan *et al*., 2011). Thus, fast sinking particles may provide microscale sub‐ and anoxic habitats supporting an autotrophic lifestyle based on sulfur‐oxidation (Karl *et al*., 1984; Shanks and Reeder, 1993). Together with *Alteromonadales* that are abundant in the North Atlantic Drift Province, these two groups seem to prefer high productivity regions with relatively fresh input from the epipelagic layer or alternatively, relatively fresh material from the formation site of the water masses. The uncultured deltaproteobacterial group Sva0853 (Supporting Information Fig. S4), belonging to the SAR324 cluster, was abundant in the bathypelagic realm as it contributed up to 20% to the communities in the AAIW and NEADW; it was also found abundantly at the Pacific Hawaii Ocean Time‐Series (HOT) station in a similar depth range (Shi *et al*., 2011). Members of the *Thiohalorhabdales*, a hitherto unknown group for marine pelagic waters, were relatively abundant in all deep‐water masses (≥2%) (Supporting Information Figs. S3 and S4). *Thiohalorhabdales* were only recently isolated from a hypersaline lake and are characterized as facultatively anaerobic and obligate sulfur‐oxidizing bacteria (Sorokin *et al*., 2008). Interestingly, we found a correlation of the relative contribution of *Thiohalorhabdales* to the total bacterial community with nitrite concentration (Fig. 5B). This supports the notion, that some of their members are denitrifiers with a possible function in the sulfur cycling of the mesopelagic layer.

## Conclusion

Our data suggest that water masses are strong predictors of the community composition in the deep North Atlantic, however, Longhurst provinces have an impact on the diversity of the bacterial community in different deep‐water masses of the North Atlantic. Probably not unexpected, the *Pelagibacteraceae* were the most abundant group in all water masses and all provinces. We found that members of the *Bacteroidetes*, *Chromatiales*, *Alteromonadales*, *Oceanospirillales* and *Gemmatimonadetes* mainly occur in the northern more productive NADR. However, toward the southern, less productive North Atlantic Tropical Gyral province the *Ricketsiales*, the SAR202‐cluster and the *Myxococcales* as well as the *Rhodospirillales* increased in relative abundance. Furthermore, our analyses indicate that the connectivity of the bacterial communities between the surface and the dark ocean is more pronounced for the mesopelagic than the bathypelagic water masses. The strength of this connectivity likely depends on the interaction between horizontal dispersal by advection, the magnitude of particle flux and the strength of water column stratification. The biogeographic distribution of the rare taxa is comparable to the more abundant groups. However, the low‐abundant taxa are more important for the overall changes in the bacterial community composition in the deep‐water masses than the more abundant taxa. Thus taken together, the dark ocean microbial biogeography is far more complex than hitherto assumed.

## Experimental procedures

### Sampling sites

During the MEDEA‐1 research cruise (October 2011), a 2,840 km long transect following the eastern branch of the North Atlantic Deep Water (NEADW) was performed from 48.75°N, 22.51°W to 26.06°N, 36.12°W crossing three biogeographic provinces as defined in Longhurst (2007): the North Atlantic Drift Province (NADR), the North Atlantic Subtropical Province (NAST) and the North Atlantic Tropical Gyral Province (NATR, Fig. 1). Satellite derived net primary production (NPP), averaged over the time of the cruise, was used to set the flexible borders of the provinces to best fit their described characteristics in Longhurst (2007). NPP rates for each sampling day were retrieved from the ocean productivity website (http://www.science.oregonstate.edu/ocean.productivity) using the Vertically Generalized Production Model (VGPM) of Behrenfeld and Falkowski (1997).

To determine the prokaryotic community composition and environmental parameters, samples at 11 stations and 6 depths ranging from 100 to 5,000 m were collected using 19 × 25 liter Niskin bottles (from Ocean Test Equipment) mounted in a rosette frame equipped with calibrated conductivity‐temperature‐depth sensors (CTD; Sea‐Bird electronic SBE911plus). The factory calibration of the conductivity sensor was checked for drift with on board measurements of salinity samples and standards using a Guildline 8400B salinometer. The deep‐water masses were identified by their salinity‐temperature characteristics and samples were collected from the epipelagic 100 m depth layer, subsequently coined subsurface layer (SSL); from the mesopelagic, the oxygen minimum layer (O_2_‐min), the Mediterranean Sea Outflow Water (MSOW) and the Antarctic Intermediate Water (AAIW) were sampled. From the bathypelagic zone, water from the North East Atlantic Deep Water (NEADW) and the Lower Deep Water (LDW) was collected. A detailed description of the physico‐chemical properties of the individual water masses encountered in our study region is given in Supporting Information Table S2.

### Environmental parameters

Oxygen concentrations were measured with an oxygen sensor (SBE43, Seabird Electronics) mounted on the CTD and calibrated daily with discrete oxygen measurements using a spectrophotometric Winkler approach (Reinthaler *et al*., 2006). Apparent oxygen utilization (AOU) was calculated as the difference between the oxygen concentration at saturation level and the observed oxygen concentration. AOU was calculated with the software package Ocean Data View (Schlitzer, 2006) using the output of the calibrated CTD and the O_2_ sensor.

Determination of inorganic nutrient concentrations followed Joint Global Ocean Fluxes Study recommendations (Gordon *et al*., 1993). On board, the concentrations of the dissolved inorganic nutrients ${\text{SiO}}_{4}^{-}$, ${\text{NO}}_{3}^{-}$, ${\text{NO}}_{2}^{-}$, ${\text{PO}}_{4}^{3-}$ were determined using an autoanalyzer (TRAACS, Technicon) immediately after collecting and filtering the samples through pre‐rinsed 0.2 µm filters (Acrodisc, Pall Corporation).

For total organic carbon (TOC) measurements, aliquots of 10 mL seawater were transferred into combusted glass ampoules. Subsequently, the pH was adjusted to <2 with concentrated phosphoric acid. Thereafter, the ampoules were sealed and stored frozen at −20°C until analysis in the lab. TOC concentrations were determined by a high temperature combustion method using a Shimadzu TOC 5000A analyzer (Benner and Strom, 1993). The TOC concentrations were calculated from quadruplicate sample injections compared to a three‐point standard curve prepared with potassium hydrogen phthalate. The instrument's performance and the validity of the calibration were determined using reference material of the Hansell consensus reference materials program (44–46 µmol L^−1^ for the reference samples; *n* = 3 and 1–2 µmol L^−1^ for low carbon water; *n* = 3). The average analytical precision of the instrument was <3%.

### Prokaryotic abundance and heterotrophic production

Prokaryotic abundance was determined as previously described (Marie *et al*., 1997). Briefly, duplicate seawater samples were fixed with electron microscopy grade glutaraldehyde (0.5% final concentration), flash‐frozen and stored at −80°C until analysis. In the home laboratory, the samples were thawed, stained with SYBR Green I (Life Technologies) in the dark for 10 min and counted on a FACS Aria II flow cytometer (BD Biosciences). Prokaryotes were analyzed on cytograms of side scatter *versus* green fluorescence and high nucleic acid containing cells (HNA) were distinguished from low nucleic acid containing cells (LNA) by gating (Gasol and Del Giorgio, 2000).

Leucine incorporation was measured as described in Reinthaler *et al*. (2010). Briefly, ³H‐leucine was added to the seawater samples to a final concentration of 5–10 nM. Following incubation at *in situ* temperature in the dark for 4–24 h, samples were filtered on to 0.2‐µm polycarbonate filters (Nuclepore, Whatman). Filters were extracted twice with 5% ice‐cold trichloroacetic acid and subsequently dried in 20 mL scintillation vials. Eight mL scintillation cocktail (FilterCount, Canberra‐Packard) was added to the samples and after 18 h, filters were counted in a liquid scintillation counter (Tricarb 3100TR, Perkin Elmer). Prokaryotic heterotrophic production was calculated from the blank‐corrected leucine incorporation rates assuming a theoretical conversion factor of 1.55 kg mol^−1^ leucine (Kirchman, 1993). A detailed description of the biological parameters of the individual water masses encountered in our study region is given in Supporting Information Table S4.

### DNA extraction and sequencing

Samples for DNA analysis were collected from six depth layers at each station. Ten liter of seawater of each depth were filtered through 0.2‐µm polycarbonate filters (47 mm diameter, Millipore). Filters were flash‐frozen in liquid nitrogen for 10 min and then stored at −80°C until further processing. Back in the lab, the filters were cut into small pieces to increase the subsequent lysis efficiency using sterile scissors. DNA from filter sections was extracted using the UltraClean Soil DNA Isolation Kit according to recommendations (MoBio). The commercial kit includes lysis, bead‐based homogenization by vortexing, and DNA cleanup with silica membranes in microfuge tubes. Tag sequencing and prior PCR‐amplification were performed at the Research and Testing Laboratory (Lubbock, TX) on a Roche 454 FLX platform using Titanium series reagents (Dowd *et al*., 2008). Primers 28F (GAGTTTGATCNTGGCTCAG) and 519R (GTNTTACNGCGGCKGCTG) were used for amplification of the variable regions V1‐3 for Bacteria (Fan *et al*., 2012). Per sample ∼1,500 sequences with an average read length of ∼370 bp were obtained. After quality filtering and denoising following Reeder and Knight (2010), the sequences were analyzed with QIIME 1.6.0 (Caporaso *et al*., 2010) using the *de novo* OTU picking workflow described in detail on the QIIME website. Adopting a 97% sequence similarity threshold, operational taxonomic units (OTUs) were assigned with the Greengenes 12.0 database using the RDP classifier implemented in QIIME. Relative OTU abundances were calculated from the obtained OTUs normalized to the total OTU counts.

Pyrotag sequences have been deposited in the National Center for Biotechnology Information (NCBI) Sequence Read Archive (SRA) under bioproject number PRJNA262973.

### Alpha‐ and beta‐diversity assessment

To constrain the diversity assessment, we applied two approaches to estimate *alpha*‐ and *beta*‐diversity by using the occurrence and relative abundance of OTUs as well as measures of sequence similarity serving as proxies for phylogenetic distinctness between communities.

The Chao1 index was used to assess OTU richness of samples. Samples were rarefied using the web‐based program iNEXT to account for varying sampling depth (Hsieh, 2013). iNEXT uses the occurrence of singletons and doubletons to estimate completeness of all the samples, identifying the sample with the lowest species coverage. Thereafter, the samples are compared at equal species completeness, which is a more sensitive approach than rarefaction based on sample size to uncover relative differences in richness between the communities (Chao and Jost, 2012).

Divergence‐based *alpha*‐diversity was determined by calculating phylogenetic diversity (PD) in QIIME (Faith, 1992). A tree was constructed using FastTree (Price *et al*., 2009) on Lane‐mask filtered sequences (Lane, 1991). The resulting sequences were aligned with the PyNAST algorithm (Caporaso *et al*., 2010). PD considers the branch lengths between different species in each sample. The PD values were compared at similar sample size using rarefaction curves.

OTU based *beta*‐diversity was analyzed in PRIMER6 (Clarke, 1993). Relative OTU abundances were square‐root transformed resulting in linear species rank‐abundance curves. Nonmetric multidimensional scaling plots (NMDS) and analyses of similarity (ANOSIM) with at least 999 permutations were performed on Bray–Curtis similarity matrices.

For divergence‐based *beta*‐diversity, weighted Unifrac analysis was used. In brief, weighted Unifrac analysis results in a similarity matrix by using comparisons of branch length differences between communities (samples) in a phylogenetic tree (Lozupone and Knight, 2005). Stronger weights are given to branches with higher relative abundances. Thus, phylogenetically more similar communities are clustered more closely together than phylogenetically more distant communities.

Additionally, linear mixed effect models (LME) were used to investigate the influence of biogeographic provinces on the dissimilarity of the deep‐water communities to its overlying SSL (for details see Supporting Information).

### Analysis of environmental influence on community composition

For analysis of environmental parameters influencing the community composition, the OTUs were phylogenetically classified. The original OTUs were clustered into groups of different taxonomic ranks including order, class and family. The result was subsequently compared to weighted Unifrac similarity using Mantel tests (Mantel, 1967). Clustering with the highest similarity to weighted Unifrac (rho = 0.87) resulted in 129 bins, which are referred to as phylotypes. To reveal the major environmental factors and their contribution to the encountered community composition variance partitioning was performed on the Hellinger‐transformed dataset using the varpart function of vegan (Oksanen *et al*., 2013).

### Additional statistical analysis

After validation of homoscedasticity, analysis of variance (ANOVA) followed by Tukey's HSD tests was used to examine significant differences between factors. If homoscedasticity was not achieved, Mann–Whitney *U* tests with Bonferroni–Holm correction were performed to determine statistically homogenous groups. Community composition and its environmental forcing was analyzed by principal component analyses (PCA) and redundancy analyses (RDA) using Hellinger‐transformed data, essentially following the recommendations of Legendre and Legendre (1998). The significance of the principal component axes was checked with the broken stick model (Frontier, 1976). Redundancy analysis can be understood as a direct extension of multiple regression to modeling of multivariate response data and as a canonical extension of principal component analysis (Legendre and Legendre, 1998). It has been argued that in ecological studies, redundancy analysis is more robust than canonical correlation analysis (CCA), especially when the appearance or disappearance of a certain taxa is of no exceptional importance for the data set (Legendre and Gallagher, 2001). One major problem in the statistical analysis with many oceanographic datasets is co‐correlation of environmental factors with depth and latitude. However, through stepwise reduction of the constraining variables, we obtained a reasonable amount of unbiased factors describing the dataset without redundancy. LME, PCA, RDA, variance partitioning, and multiple single factor analyses were carried out using R 2.15.0 (R Core Team, 2013). For details on the used packages see the supplementary information.

## Supporting information

Additional Supporting Information may be found in the online version of this article at the publisher's web‐site:

**Table S1.** Diversity indices from different water mass and biogeographic province
**Table S2.** Mean physico‐chemical parameters of the water masses and biogeographical provinces.
**Table S3.** Statistics of rare, abundant and common bacteria
**Table S4.** Mean biological parameters of the water masses and biogeographical provinces.
**Table S5.** Probe sequences and formamide concentrations used for CARD‐FISH
**Fig. S1.** Shared and unique OTUs in the different pelagic realms.
**Fig. S2.** Rarefaction and coverage plots the richness estimator Chao1
**Fig. S3.** Water mass‐specific bacterial phylotypes.
**Fig. S4.** Heat‐plot of all bacterial groups in the different water masses
**Fig. S5.** NMDS of the different bacterial abundance classes.
