# Supplementary material for: Connectivity between surface and deep waters determines prokaryotic diversity in the North Atlantic Deep Water
Source: Environ Microbiol. 2016 Mar 30;18(6):2052–63. doi: 10.1111/1462-2920.13237 (PMC4921061; doi:10.1111/1462-2920.13237)
Supplement: Supplementary file 1 — Table S1. Diversity indices from different water mass and biogeographic province Table S2. Mean physico‐chemical parameters of the water masses and biogeographical provinces. Table S3. Statistics of rare, abundant and common bacteria Table S4. Mean biological parameters of the water masses and biogeographical provinces. Table S5. Probe sequences and formamide concentrations used for CARD‐FISH Fig. S1. Shared and unique OTUs in the different pelagic realms. Fig. S2. Rarefaction and coverage plots the richness estimator Chao1 Fig. S3. Water mass‐specific bacterial phylotypes. Fig. S4. Heat‐plot of all bacterial groups in the different water masses Fig. S5. NMDS of the different bacterial abundance classes. [file EMI-18-2052-s001.pdf]

**Supplementary Information**

Connectivity between surface and deep waters determines prokaryotic diversity in the North Atlantic Deep Water

Alexander H. Frank, Juan A. Garcia, Gerhard J. Herndl, Thomas Reinthaler

**Supplementary Methods**

**Linear mixed effect models**

**CARD-FISH procedure**

**R packages used**

**Evaluation of the sequencing approach**

**Supplementary Tables**

Table S1 **Diversity indices from different water mass and biogeographic province**

Table S2 **Mean physico-chemical parameters of the water masses and biogeographical provinces.**

Table S3 **Statistics of rare, abundant and common bacteria**

Table S4 **Mean biological parameters of the water masses and biogeographical provinces.**

Table S5 **Probe sequences and formamide concentrations used for CARD-FISH**

**Supplementary Figures**

Figure S1 **Shared and unique OTUs in the different pelagic realms.**

Figure S2 **Rarefaction and coverage plots the richness estimator Chao1**

Figure S3 **Water mass-specific bacterial phylotypes.**

Figure S4 **Heat-plot of all bacterial groups in the different water masses**

Figure S5 **NMDS of the different bacterial abundance classes.**

**Supplementary References**

## Methods

### Linear Mixed Effect Model

Many common statistical models can be expressed as linear models that incorporate both fixed effects (i.e., parameters associated with certain repeatable levels of experimental factors), and random effects (i.e., parameters associated with individual experimental units drawn randomly from a population). A model with both, fixed and random effects is called a mixed-effects model (Pinheiro, J.C. and Bates, D.M., 2002). Mixed-effects models can describe relationships between a response variable and some covariates in data that are grouped according to one or more classification factors (e.g. longitudinal data, repeated measures, multilevel and block designs). By associating common random effects to observations sharing the same level of a classification factor, mixed-effects models flexibly represent the covariance structure induced by the grouping of the data (Pinheiro, J.C. and Bates, D.M., 2002). In our case, we hypothesized, that the effect of the surface community composition on the deep-water masses was dependent on the water mass (W) and the province (P), while each station (S) was treated as introducing random effects; denoted as 1|S in the function lmer of the R-package lme4 (Bates, D. *et al.*, 2013). Specifically, we extracted the weighted UniFrac dissimilarity ($D_{wu}$) between the subsurface layer (SSL at ~100 m depth) and each sample below at the same station (n = 5).
Firstly, we built a linear mixed effect model (lm) where province (P) was the fixed and the station (S) was the random effect on $D_{wu}$, obtaining our first hypothetical model $A_H$:

$$A_H = \text{lm}\ (D_{wu} \sim P + (1|S)) \qquad (1)$$

A simple null model ($A_0$) was introduced that omits the effect of the provinces:

$$A_0 = \text{lm}\ (D_{wu} \sim (1|S)) \qquad (2)$$

Subsequently the full model and the null model were tested against each other. The test was significant ($\text{Chi}^2_2 = 8.02$, $p < 0.05$) for our data set.
Secondly, a model ($B_H$) was introduced, which assumed an interaction between water mass (W) and province (P) influencing the effect of the surface community on the deep-water community:

$$B_H = \text{lm}\ (D_{wu} \sim W * P + (1|S)) \qquad (3)$$

$B_H$ was tested against $A_H$, and as expected, a highly significant effect of the water mass was found ($\text{Chi}^2_{12} = 65.9$, $p < 0.001$).

To examine the effect of province on the dissimilarity between the deep waters and the SSL we formulated:

$$C_H = \text{lm}\ (D_{wu} \sim W + (1|S)) \qquad (4)$$

Finally we tested $C_H$ against $B_H$. The result was highly significant ($\text{Chi}^2_{10} = 27.0$, $p < 0.01$) indicating a significant effect of province to explain the dissimilarity of the water mass to the SSL.

## Fixation, preparation, and analysis of CARD-FISH samples

CARD-FISH was used to determine the abundance of major groups of prokaryotes at nine stations (Stns 5, 6, 7, 9, 10, 11, 13, 14, and 15) along the transect, essentially following the method described by Pernthaler, A. *et al.* (2002). Immediately after collecting the samples from the Niskin bottles, 20–80 mL of water were fixed by adding formaldehyde (37%; 2% final concentration) and stored at 4°C in the dark. After 18 h, the samples were filtered through 0.2-µm polycarbonate filters (Millipore GTTP, 25 mm filter diameter) supported by nitrate cellulose filters (Millipore, HAWP, 0.45 µm), washed twice with 10 mL Milli-Q water, dried and stored in a microfuge vial at -20°C until further processing in the laboratory. For hybridization we used horseradish peroxidase-labeled oligonucleotide probes to determine the fraction of 4',6-diamidino-2-phenylindole (DAPI)-stained cells. The hybridization conditions and probes targeting Bacteria, SAR11 (Alphaproteobacteria) and SAR202 (Chloroflexi), are given in Supplementary Table S4. Cells were counter-stained with a DAPI-mix [5.5 parts of Citifluor (Citifluor), 1 part of Vectashield (Vector Laboratories) and 0.5 parts of phosphate-buffered saline (PBS) with DAPI (final concentration 1 µg $mL^{-1}$)]. Enumeration of DAPI-stained cells and cells stained with the specific probes was performed with a Zeiss Axioplan 2 epifluorescence microscope equipped with a 100-W Hg lamp and appropriate filter sets for DAPI, and Alexa448. A minimum of 600 DAPI-stained cells was counted per sample. An AxioCam MRm black and white camera (Carl Zeiss, Germany) was used to capture pictures using a plan apochromatic 100x/1.46 oil objective. To obtain relative abundances comparable to the 454-data, the number of SAR11 and SAR202 probe-positive cells was divided by the EUB-positive cells of the same sample.

## R packages used for the analysis of datasets

The statistical tests used to analyze the data were performed in R with the help of the following freely available packages: Ade4 (Dray and Dufour, 2007), Agricolae (de Mendiburu, 2013), FactoMineR (Husson *et al.*, 2013), MASS (Venables and Ripley, 2002), sna (Butts, 2013), Vegan (Oksanen *et al.*, 2013) and Venneuler (Wilkinson, 2011).

## Evaluation of the 454 sequencing approach

Commonly, three-percent sequence dissimilarity in the 16S rRNA gene is used as a threshold to delineate operational taxonomic units (OTUs) (Martin, 2002) although this might not be appropriate for all prokaryotes and likely masks a considerable amount of micro-diversity (Garcia-Martinez and Rodriguez-Valera, 2000; Acinas *et al.*, 2004). Sequencing the V1–V3 region has been shown to yield similar phylogenetic affiliations compared to Sanger sequencing (Kumar *et al.*, 2011) and the region was found better for phylogenetic community characterization when compared to short sequences of the hypervariable V6 region used in the past (Liu *et al.*, 2007; Jeraldo *et al.*, 2011). However, to check the consistency of 454 pyrosequencing-derived relative OTU abundances, we performed a comparison with quantitative CARD-FISH analysis using a set of SAR11 and SAR202 probes (see Supplementary Table S5 and Supplementary Figure S6). This comparison yielded a good agreement between both methods similarly to that recently reported by Logares *et al.* (2013). Based on these methodological issues, however, the explanatory power of commonly used diversity indices remains uncertain (Tuomisto, 2010).

## Tables

**Table S1** Diversity indices from different water masses and biogeographical provinces

| WM | Province | Bac Seq[a] | Bac OTU[b] | Chao1[c] | PD[d] |
|---|---|---|---|---|---|
| **SSL** | | **814 ± 284** | **130 ± 36** | **63 ± 17** | **8.99 ± 1.36** |
| | NADR | 665 ± 475 | 116 ± 55 | 60 ± 21 | 9.74 ± 1.33 |
| | NAST | 928 ± 152 | 154 ± 27 | 71 ± 22 | 9.55 ± 1.24 |
| | NATR | 849 ± 135 | 120 ± 12 | 57 ± 9 | 7.69 ± 0.49 |
| **$O_2$-min** | | **804 ± 229** | **180 ± 23** | **115 ± 19** | **10.95 ± 0.45** |
| | NADR | 696 ± 175 | 157 ± 17 | 98 ± 9 | 10.95 ± 0.81 |
| | NAST | 1050 ± 296 | 198 ± 28 | 103 ± 10 | 10.64 ± 0.02 |
| | NATR | 712 ± 114 | 184 ± 14 | 133 ± 10 | 11.15 ± 0.36 |
| **MSOW** | | **821 ± 260** | **189 ± 42** | **122 ± 23** | **11.45 ± 0.62** |
| | NADR | 713 ± 320 | 155 ± 29 | 101 ± 10 | 11.34 ± 0.7 |
| | NAST | 1014 ± 295 | 221 ± 52 | 127 ± 15 | 11.54 ± 1.06 |
| | NATR | 792 ± 43 | 207 ± 1 | 149 ± 3 | 11.52 ± 0.34 |
| **AAIW** | | **741 ± 112** | **169 ± 20** | **106 ± 21** | **11.62 ± 0.97** |
| | NADR | 801 ± 182 | 162 ± 24 | 91 ± 4 | 10.94 ± 0.69 |
| | NAST | 707 ± 81 | 159 ± 18 | 95 ± 13 | 11.4 ± 1.14 |
| | NATR | 713 ± 51 | 187 ± 9 | 133 ± 1 | 12.52 ± 0.14 |
| **NEADW** | | **664 ± 136** | **159 ± 18** | **106 ± 17** | **12.22 ± 0.99** |
| | NADR | 694 ± 103 | 153 ± 8 | 91 ± 4 | 11.59 ± 0.41 |
| | NAST | 756 ± 111 | 169 ± 11 | 108 ± 3 | 12.77 ± 0.86 |
| | NATR | 541 ± 117 | 154 ± 30 | 118 ± 25 | 12.29 ± 1.39 |
| **LDW** | | **551 ± 107** | **127 ± 24** | **82 ± 18** | **11.94 ± 1.09** |
| | NADR | 526 ± 74 | 115 ± 5 | 71 ± 8 | 11.48 ± 1.08 |
| | NAST | 526 ± 154 | 121 ± 27 | 78 ± 14 | 11.9 ± 1.53 |
| | NATR | 599 ± 106 | 145 ± 29 | 97 ± 23 | 12.43 ± 0.76 |

For abbreviation of water masses and biographical provinces see Supplementary Table S1.
[a]Bac Seq: bacterial sequences after trimming and quality filtering
[b]Bac OTU: Operational taxonomic units at 97% similarity thresholds
[c]Chao 1: Richness estimator Chao 1 rarefied to base coverage (lowest covered sample = 80%)
[d]PD: Phylogenetic diversity (Faith, 1992) at lowest sample size (rarefied)
Numbers indicate averages ± standard deviations (n = 3 per province)

**Table S2** Physico-chemical parameters in the water masses (WM) and biogeographical provinces of the transect in the North East Atlantic

| WM[a] | Province[b] | Depth (m) | Theta (°C) | Salinity | Si (µmol/kg) | $O_2$ (µmol/kg) | $PO_4$ (µmol/kg) | $NO_3$ (µmol/kg) | $NO_2$ (µmol/kg) | TOC (µmol/kg) |
|---|---|---|---|---|---|---|---|---|---|---|
| **SSL** | | **98 ± 5** | **16.86 ± 3.37** | **36.34 ± 0.53** | **1.4 ± 0.9** | **226 ± 5** | **0.21 ± 0.23** | **3.4 ± 3.9** | **0.048±0.045** | **64 ± 10** |
| | NADR | 100 ± 1 | 13.12 ± 0.79 | 35.76 ± 0.14 | 2.5 ± 0.5 | 231 ± 3 | 0.49 ± 0.12 | 8.1 ± 1.8 | 0.045±0.009 | 67 ± 3 |
| | NAST | 96 ± 8 | 17.10 ± 1.95 | 36.36 ± 0.26 | 1.1 ± 0.4 | 222 ± 3 | 0.12 ± 0.12 | 2.0 ± 2.0 | 0.086±0.064 | 64 ± 18 |
| | NATR | 100 ± 1 | 20.36 ± 1.25 | 36.90 ± 0.27 | 0.6 ± 0.1 | 225 ± 5 | 0.01 ± 0.00 | 0.0 ± 0.0 | 0.014±0.005 | 62 ± 4 |
| **$O_2$-min** | | **783 ± 138** | **9.67 ± 0.83** | **35.47 ± 0.21** | **10.2 ± 1.9** | **175 ± 9** | **1.20 ± 0.15** | **19.4 ± 2.2** | **0.022±0.004** | **53 ± 7** |
| | NADR | 792 ± 256 | 9.46 ± 0.98 | 35.49 ± 0.35 | 9.6 ± 0.6 | 183 ± 4 | 1.14 ± 0.10 | 18.3 ± 1.2 | 0.022±0.005 | 55 ± 2 |
| | NAST | 732 ± 50 | 10.38 ± 0.15 | 35.54 ± 0.11 | 8.9 ± 0.2 | 177 ± 3 | 1.11 ± 0.03 | 18.3 ± 0.6 | 0.023±0.003 | 56 ± 6 |
| | NATR | 826 ± 24 | 9.18 ± 0.78 | 35.37 ± 0.12 | 12.1 ± 2.4 | 166 ± 9 | 1.34 ± 0.17 | 21.7 ± 2.5 | 0.019±0.005 | 48 ± 10 |
| **MSOW** | | **1024 ± 111** | **8.32 ± 1.42** | **35.53 ± 0.23** | **11.3 ± 1.6** | **194 ± 16** | **1.17 ± 0.10** | **18.8 ± 1.4** | **0.022±0.004** | **56 ± 10** |
| | NADR | 972 ± 124 | 8.39 ± 2.49 | 35.53 ± 0.37 | 9.9 ± 0.9 | 202 ± 29 | 1.11 ± 0.08 | 17.8 ± 0.7 | 0.023±0.004 | 61 ± 11 |
| | NAST | 1066 ± 59 | 8.60 ± 0.62 | 35.64 ± 0.16 | 11.4 ± 0.5 | 192 ± 3 | 1.14 ± 0.07 | 18.5 ± 1.0 | 0.019±0.001 | 53 ± 8 |
| | NATR | 1033 ± 153 | 7.98 ± 1.09 | 35.42 ± 0.10 | 12.5 ± 1.9 | 189 ± 6 | 1.26 ± 0.09 | 20.2 ± 1.2 | 0.023±0.006 | 55 ± 14 |
| **AAIW** | | **1530 ± 156** | **4.98 ± 0.71** | **35.14 ± 0.11** | **13.1 ± 2.7** | **239 ± 12** | **1.19 ± 0.07** | **18.6 ± 1.0** | **0.019±0.003** | **51 ± 11** |
| | NADR | 1364 ± 115 | 5.08 ± 1.08 | 35.10 ± 0.16 | 10.8 ± 0.1 | 245 ± 18 | 1.15 ± 0.01 | 17.9 ± 0.2 | 0.021±0.003 | 58 ± 9 |
| | NAST | 1533 ± 58 | 5.16 ± 0.79 | 35.19 ± 0.12 | 12 ± 0.4 | 240 ± 11 | 1.15 ± 0.01 | 18.2 ± 0.1 | 0.019±0.002 | 52 ± 7 |
| | NATR | 1693 ± 12 | 4.69 ± 0.17 | 35.14 ± 0.03 | 16.4 ± 1.6 | 232 ± 7 | 1.27 ± 0.07 | 19.7 ± 0.9 | 0.016±0.003 | 41 ± 10 |
| **NEADW** | | **2749 ± 2** | **2.75 ± 0.10** | **34.95 ± 0.01** | **25.5 ± 5.4** | **254 ± 8** | **1.29 ± 0.09** | **19.5 ± 1.2** | **0.021±0.003** | **49 ± 10** |
| | NADR | 2749 ± 3 | 2.82 ± 0.10 | 34.94 ± 0.01 | 19.5 ± 2.7 | 263 ± 4 | 1.20 ± 0.03 | 18.1 ± 0.5 | 0.022±0.002 | 56 ± 5 |
| | NAST | 2750 ± 1 | 2.71 ± 0.12 | 34.95 ± 0.01 | 26.4 ± 3.6 | 253 ± 3 | 1.29 ± 0.05 | 19.6 ± 0.6 | 0.021±0.003 | 51 ± 10 |
| | NATR | 2747 ± 1 | 2.72 ± 0.03 | 34.96 ± 0.00 | 30.5 ± 2 | 245 ± 4 | 1.38 ± 0.05 | 20.7 ± 0.6 | 0.018±0.003 | 41 ± 9 |
| **LDW** | | **4304 ± 334** | **2.13 ± 0.11** | **34.91 ± 0.01** | **43.3 ± 2.0** | **244 ± 2** | **1.49 ± 0.01** | **22.3 ± 0.3** | **0.019±0.004** | **47 ± 6** |
| | NADR | 4039 ± 58 | 2.23 ± 0.02 | 34.92 ± 0.00 | 41.7 ± 0.8 | 241 ± 1 | 1.48 ± 0.01 | 22.1 ± 0.2 | 0.022±0.003 | 52 ± 3 |
| | NAST | 4170 ± 60 | 2.15 ± 0.03 | 34.91 ± 0.00 | 42.8 ± 0.7 | 244 ± 1 | 1.48 ± 0.01 | 22.3 ± 0.1 | 0.018±0.003 | 49 ± 5 |
| | NATR | 4701 ± 265 | 2.00 ± 0.08 | 34.90 ± 0.01 | 45.5 ± 1.6 | 247 ± 1 | 1.50 ± 0.01 | 22.6 ± 0.2 | 0.017±0.004 | 43 ± 7 |

[a]Water masses: SSL – Subsurface Layer (100m), $O_2$-min – $O_2$ Minimum, MSOW – Mediterranean Sea Outflow Water, AAIW – Antarctic Intermediate Water, NEADW – North East Atlantic Deep Water, LDW – Lower Deep Water.
[b]Provinces: NADR – North Atlantic Drift Province, NAST – North Atlantic Subtropical Province, NATR – North Atlantic Tropical Gyral Province
Numbers indicate averages ± standard deviations (n = 3 per province)

**Table S3** ANOSIM (global R) and Mantel (weighted Spearman rho) statistics (all p < 0.00l) to test the influence of the abundance fractions on the differences in community composition between provinces and water masses in the meso- and bathypelagic waters.

| ANOSIM | Rare | Common | Abundant | All |
|---|---|---|---|---|
| Province [a] | 0.512 | 0.518 | 0.442 | 0.558 |
| Water mass [b] | 0.647 | 0.763 | 0.722 | 0.803 |
| Ratio (a/b) | 0.79 | 0.69 | 0.61 | 0.69 |

| Mantel | Rare | Common | Abundant | All |
|---|---|---|---|---|
| Rare | 1 | 0.832 | 0.7 | 0.902 |
| Common | | 1 | 0.787 | 0.926 |
| Abundant | | | 1 | 0.825 |
| All | | | | 1 |

[a]Fractions rare (< 1% of the community), abundant (1–10 % of the community) and common (> 10% of the community) as defined in the main text.

**Table S4** Biological parameters according to water mass (WM) and biogeographical provinces

| WM | Province | AOU (µmol $L^{-1}$) | FLC (ng Chla $L^{-1}$) | VA ($10^6$ $ml^{-1}$) | PA ($10^5$ $ml^{-1}$) | Leu inc (pmol $L^{-1}$ $h^{-1}$) | PHP (µmol C $m^{-3}$ $d^{-1}$) |
|---|---|---|---|---|---|---|---|
| **SSL** | | **17 ± 13** | **99 ± 48** | **2.69 ± 0.73** | **3.34 ± 0.63** | **3.2654 ± 3.3593** | **10.1229 ± 10.4138** |
| | NADR | 28.0 ± 2.6 | 28.4 ± 6.7 | 2.297 ± 0.899 | 3.89 ± 0.91 | 1.1283 ± 0.2439 | 3.4977 ± 0.7562 |
| | NAST | 19.3 ± 10.3 | 127.6 ± 18.5 | 3.004 ± 0.689 | 2.94 ± 0.44 | 1.8632 ± 1.3155 | 5.7759 ± 4.0779 |
| | NATR | -0.2 ± 5.6 | 113 ± 33.2 | 2.434 ± 0.815 | 3.59 ± 0.20 | 8.2071 ± 2.8113 | 25.442 ± 8.7149 |
| **$O_2$-min** | | **104 ± 9** | **15 ± 2** | **0.31 ± 0.17** | **0.80 ± 1.12** | **0.0486 ± 0.0349** | **0.1505 ± 0.1083** |
| | NADR | 96.9 ± n.a. | 17.2 ± n.a. | 0.560 ± n.a. | 2.76 ± n.a. | 0.1078 ± n.a. | 0.3341 ± n.a. |
| | NAST | 99 ± 4.1 | 15.6 ± 0.9 | 0.285 ± 0.174 | 0.48 ± 0.26 | 0.0433 ± 0.0048 | 0.1342 ± 0.0148 |
| | NATR | 112.6 ± 9.6 | 12.2 ± 1.3 | 0.209 ± 0.036 | 0.13 ± 0.07 | 0.0242 ± 0.0107 | 0.075 ± 0.0330 |
| **MSOW** | | **96 ± 11** | **14 ± 2** | **0.33 ± 0.28** | **0.64 ± 0.56** | **0.0390 ± 0.0288** | **0.1210 ± 0.0892** |
| | NADR | 88.0 ± 12.1 | 14.1 ± 2.8 | 0.618 ± 0.132 | 1.04 ± 0.70 | 0.0657 ± 0.0214 | 0.2035 ± 0.0663 |
| | NAST | 96.6 ± 5.5 | 13.2 ± 1.3 | 0.127 ± 0.026 | 0.32 ± 0.02 | 0.0242 ± 0.0116 | 0.0749 ± 0.0360 |
| | NATR | 106.4 ± 0 | 13.1 ± 0.1 | 0.097 ± 0.004 | 0.35 ± 0.09 | 0.014 ± 0.0098 | 0.0434 ± 0.0305 |
| **AAIW** | | **77 ± 10** | **12 ± 2** | **0.39 ± 0.39** | **0.43 ± 0.38** | **0.0171 ± 0.0135** | **0.0531 ± 0.0419** |
| | NADR | 69.0 ± 10.2 | 13.1 ± 3.1 | 0.872 ± 0.228 | 0.86 ± 0.39 | 0.0341 ± 0.0061 | 0.1058 ± 0.0189 |
| | NAST | 74.4 ± 5.1 | 11.9 ± 1.3 | 0.215 ± 0.096 | 0.22 ± 0.07 | 0.0117 ± 0.0021 | 0.0363 ± 0.0064 |
| | NATR | 86.5 ± 7 | 9.8 ± 1.0 | 0.092 ± 0.058 | 0.22 ± 0.06 | 0.0055 ± 0.0033 | 0.0171 ± 0.0102 |
| **NEADW** | | **83 ± 9** | **3 ± 2** | **0.33 ± 0.26** | **0.26 ± 0.25** | **0.0062 ± 0.0037** | **0.0193 ± 0.0116** |
| | NADR | 73.0 ± 4.3 | 2.6 ± 1.5 | 0.641 ± 0.129 | 0.51 ± 0.31 | 0.0104 ± 0.0026 | 0.0322 ± 0.0081 |
| | NAST | 84.4 ± 3.9 | 3.6 ± 3.0 | 0.255 ± 0.021 | 0.15 ± 0.02 | 0.0058 ± 0.0010 | 0.0181 ± 0.0030 |
| | NATR | 92.8 ± 4.6 | 4.1 ± 2.5 | 0.088 ± 0.035 | 0.11 ± 0.02 | 0.0024 ± 0.0004 | 0.0075 ± 0.0012 |
| **LDW** | | **103 ± 1** | **4 ± 2** | **0.29 ± 0.26** | **0.16 ± 0.12** | **0.0066 ± 0.0075** | **0.0206 ± 0.0234** |
| | NADR | 102.9 ± 0.9 | 3.1 ± 2.5 | 0.596 ± 0.203 | 0.28 ± 0.16 | 0.0148 ± 0.0086 | 0.0457 ± 0.0267 |
| | NAST | 102.0 ± 0.8 | 3.2 ± 0.8 | 0.191 ± 0.082 | 0.11 ± 0.02 | 0.0037 ± 0.0012 | 0.0115 ± 0.0037 |
| | NATR | 102.9 ± 0.5 | 5.0 ± 3.7 | 0.093 ± 0.023 | 0.09 ± 0.05 | 0.0015 ± 0.0001 | 0.0045 ± 0.0004 |

For abbreviation of water masses and biographical provinces see Supplementary Table S1.
Numbers indicate mean ± standard deviations (n > 5 per province)

**Table S5** Probe sequences and formamide concentrations used for CARD-FISH

| Probe | Sequence (5'→3') | FA[a] (%) | Reference |
|---|---|---|---|
| EUB338 I | GCTGCCTCCCGTAGGAGT | 55 | (Amann *et al.*, 1990) |
| EUB338 II | GCAGCCACCCGTAGGTGT | 55 | (Daims *et al.*, 1999) |
| EUB338 III | GCTGCCACCCGTAGGTGT | 55 | (Daims *et al.*, 1999) |
| SAR11-152R | ATTAGCACAAGTTTCCYCGTGT | 45 | (Morris *et al.*, 2002) |
| SAR11-441R | TACAGTCATTTTCTTCCCCGAC | 45 | (Morris *et al.*, 2002) |
| SAR11-542R | TCCGAACTACGCTAGGTC | 45 | (Morris *et al.*, 2002) |
| SAR11-732R | GTCAGTAATGATCCAGAAAGYTG | 45 | (Morris *et al.*, 2002) |
| SAR202-104R | GTTACTCAGCCGTCTGCC | 35 | (Morris *et al.*, 2004) |
| SAR202-312R | TGTCTCAGTCCCCCTCTG | 40* | (Morris *et al.*, 2004) |

[a]Formamide (FA) concentration in percent of hybridization buffer
*Applied formamide concentration according to Schattenhofer et al. (2009)

**Figures**

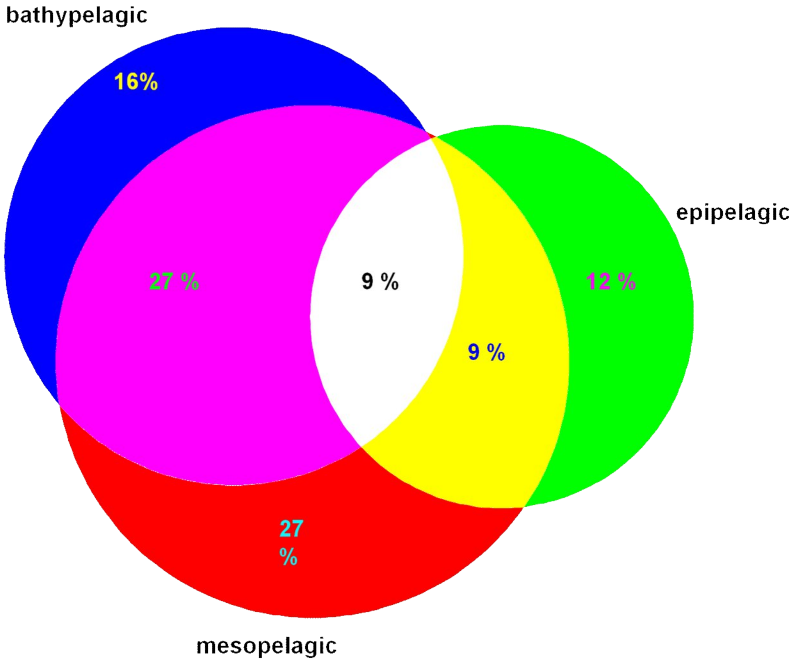


**Figure S1** Percentages of shared and unique OTUs between different pelagic realms represented as Venn diagram. The size of each area is corresponding to the percentage. Epipelagic (green) = SSL; mesopelagic (red) = $O_2$-min, MSOW, and AAIW; bathypelagic (blue) = NEADW and LDW.

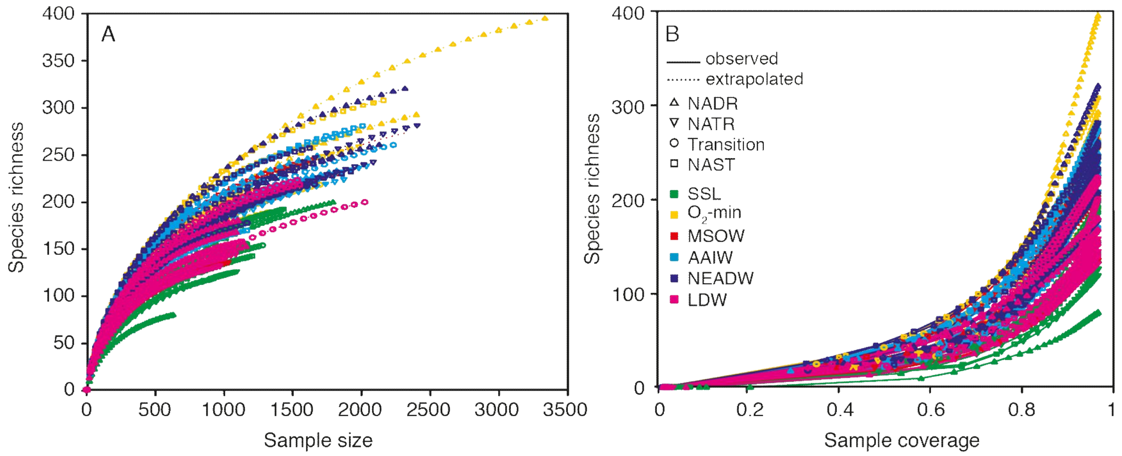


**Figure S2** Rarefaction and extrapolation curves of the Chao1 richness estimator versus sample size (A) and sample coverage (B). The sample coverage was derived from the slope of the species accumulation curves as suggested in Chao *et al.* (2012) using the program iNEXT with 200 bootstraps. The calculated minimal observed coverage of 0.8 was used for downstream statistical analysis and comparison of the Chao1 richness estimators between samples, provinces and water masses.

| Phylotype | Water mass | Mean relative abundance (%) | ordered subgroups → low | | | | high |
|---|---|---|---|---|---|---|---|
| Actinobacteria | SSL | 6.1 | | | ■ | | |
| | $O_2$-min | 2.6 | | ■ | | | |
| | MSOW | 2.6 | | ■ | | | |
| | AAIW | 1.4 | ■ | ■ | | | |
| | NEADW | 1.0 | ■ | | | | |
| | LDW | 2.2 | ■ | ■ | | | |
| Bacteroidetes | SSL | 5.0 | | | ■ | | |
| | $O_2$-min | 0.5 | ■ | | | | |
| | MSOW | 1.0 | ■ | | | | |
| | AAIW | 0.6 | ■ | | | | |
| | NEADW | 0.5 | ■ | | | | |
| | LDW | 1.9 | ■ | ■ | | | |
| Desulfobacterales | SSL | 0.6 | ■ | | | | |
| | $O_2$-min | 1.8 | | ■ | | | |
| | MSOW | 1.4 | ■ | ■ | | | |
| | AAIW | 1.5 | ■ | ■ | | | |
| | NEADW | 1.6 | ■ | ■ | | | |
| | LDW | 1.4 | ■ | ■ | | | |
| other Rickettsiales | SSL | 1.3 | ■ | | | | |
| | $O_2$-min | 0.9 | ■ | | | | |
| | MSOW | 1.2 | ■ | ■ | | | |
| | AAIW | 1.5 | ■ | ■ | ■ | | |
| | NEADW | 2.9 | | ■ | ■ | | |
| | LDW | 3.0 | | | ■ | | |
| Pelagibacteraceae | SSL | 62.7 | | | | | ■ |
| | $O_2$-min | 60.3 | | | | ■ | ■ |
| | MSOW | 54.8 | | | ■ | ■ | |
| | AAIW | 49.2 | | ■ | ■ | | |
| | NEADW | 42.6 | ■ | ■ | | | |
| | LDW | 39.2 | ■ | | | | |
| Planctomycetes | SSL | 0.4 | ■ | | | | |
| | $O_2$-min | 0.7 | ■ | ■ | | | |
| | MSOW | 0.9 | ■ | ■ | | | |
| | AAIW | 0.6 | ■ | ■ | | | |
| | NEADW | 0.8 | | ■ | | | |
| | LDW | 2.5 | | | ■ | | |

| Type | Water mass | Mean relative abundance (%) | ordered subgroups → low | | | high |
|---|---|---|---|---|---|---|
| Rhodospirillales | SSL | 0.5 | ■ | | | |
| | $O_2$-min | 1.9 | | ■ | | |
| | MSOW | 1.6 | | ■ | | |
| | AAIW | 2.1 | | ■ | | |
| | NEADW | 2.6 | | ■ | | |
| | LDW | 2.7 | | ■ | | |
| SAR202 | SSL | 0.3 | ■ | | | |
| | $O_2$-min | 3.0 | | ■ | | |
| | MSOW | 3.8 | | ■ | ■ | |
| | AAIW | 6.2 | | | ■ | ■ |
| | NEADW | 8.8 | | | | ■ |
| | LDW | 8.6 | | | | ■ |
| SAR406 | SSL | 2.1 | ■ | | | |
| | $O_2$-min | 5.0 | | ■ | | |
| | MSOW | 6.3 | | ■ | ■ | |
| | AAIW | 6.6 | | ■ | ■ | |
| | NEADW | 8.3 | | | ■ | |
| | LDW | 8.6 | | | ■ | |
| Sva0853 | SSL | 2.5 | ■ | | | |
| | $O_2$-min | 13.4 | | ■ | | |
| | MSOW | 13.5 | | ■ | | |
| | AAIW | 17.6 | | | ■ | |
| | NEADW | 17.4 | | | ■ | |
| | LDW | 13.5 | | ■ | | |
| Thiohalorhabdales | SSL | 1.2 | ■ | | | |
| | $O_2$-min | 3.4 | | ■ | ■ | |
| | MSOW | 4.5 | | | ■ | |
| | AAIW | 3.3 | | ■ | ■ | |
| | NEADW | 2.4 | ■ | ■ | | |
| | LDW | 1.9 | ■ | | | |

**Figure S3** Water mass-dependent distribution of the dominant bacterial phylotypes in the NADR, NAST and NATR. Black squares of non-overlapping ordered subgroups represent statistically significant differences at $p < 0.05$. The squares also indicate an increase or decrease in relative abundance between the water masses.

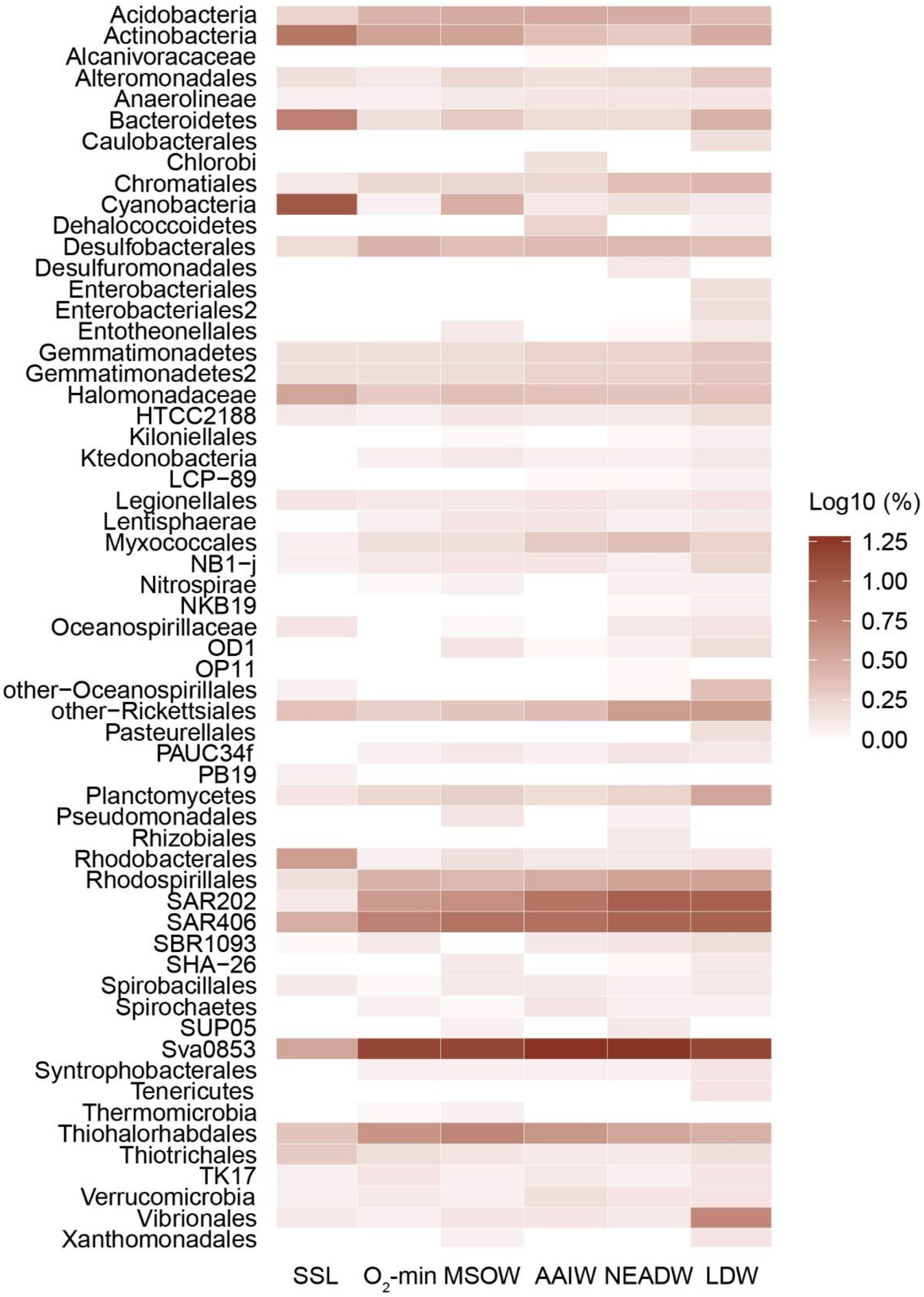
Acidobacteria
Actinobacteria
Alcanivoracaceae
Alteromonadales
Anaerolineae
Bacteroidetes
Caulobacterales
Chlorobi
Chromatiales
Cyanobacteria
Dehalococcoidetes
Desulfobacterales
Desulfuromonadales
Enterobacteriales
Enterobacteriales2
Entotheonellales
Gemmatimonadetes
Gemmatimonadetes2
Halomonadaceae
HTCC2188
Kiloniellales
Ktedonobacteria
LCP−89
Legionellales
Lentisphaerae
Myxococcales
NB1−j
Nitrospirae
NKB19
Oceanospirillaceae
OD1
OP11
other−Oceanospirillales
other−Rickettsiales
Pasteurellales
PAUC34f
PB19
Planctomycetes
Pseudomonadales
Rhizobiales
Rhodobacterales
Rhodospirillales
SAR202
SAR406
SBR1093
SHA−26
Spirobacillales
Spirochaetes
SUP05
Sva0853
Syntrophobacterales
Tenericutes
Thermomicrobia
Thiohalorhabdales
Thiotrichales
TK17
Verrucomicrobia
Vibrionales
Xanthomonadales
SSL
O2-min
MSOW
AAIW
NEADW
LDW
Log10 (%)
1.25
1.00
0.75
0.50
0.25
0.00

**Figure S4** Heatmap of all OTUs including those that were not significantly different between water masses. Original relative abundances in percent are rescaled to log10 for better visibility.

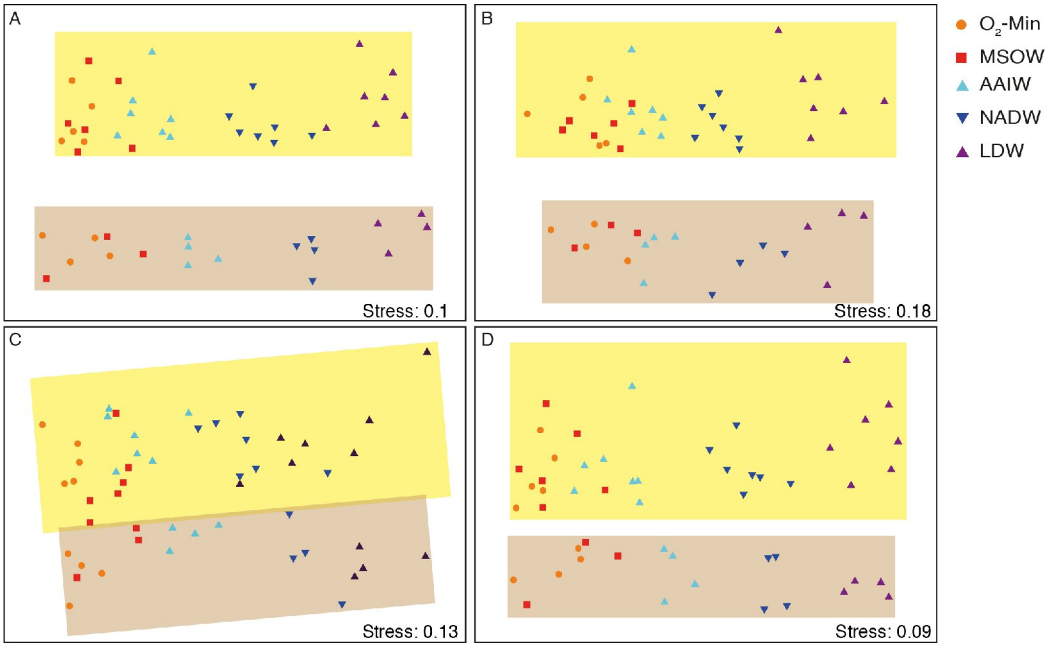


**Figure S5** Non-metric multidimensional scaling (NMDS) plots of the meso- and bathypelagic bacterial community using different abundance categories calculated as Bray-Curtis similarity on square-root transformed relative abundances of OTUs. Full range of OTUs (A), rare OTUs with abundances <1% (B), abundant OTUs occurring >10% (C), and common OTUs in the range of 1 – 10% (D). Yellow background indicates the North Atlantic Drift Province (NADR) and North Atlantic Subtropical Province (NAST), brown background color indicates the North Atlantic Tropical Gyral Province (NAST).

## References


Amann RI, Binder BJ, Olson RJ, Chisholm SW, Devereux R, Stahl, DA (1990) Combination of 16S rRNA-targeted oligonucleotide probes with flow cytometry for analyzing mixed microbial populations. *Appl Environ Microbiol* **56**: 1919-1925.

Bates D, Maechler M, Bolker, B. (2013) lme4: Linear mixed-effects models using S4 classes. R package version 0.999999-2.

Butts CT (2013) sna: Tools for social network analysis. R package version 2.3-1.

Daims H, Brühl A, Amann R, Schleifer K-H, Wagner, M. (1999) The Domain-specific probe EUB338 is insufficient for the detection of all Bacteria: Development and evaluation of a more comprehensive probe set. *Syst Appl Microbiol* **22**: 434-444.

de Mendiburu F. (2013) agricolae: Statistical Procedures for Agricultural Research. R package version 1.1-4.

Dray S, Dufour AB. (2007) The ade4 package: implementing the duality diagram for ecologists. **22**: 1-20.

Husson F, Josse J, Le S, Mazet J. (2013) FactoMineR: Multivariate Exploratory Data Analysis and Data Mining with R. R package version 1.25.

Morris RM, Rappe MS, Connon SA, Vergin KL, Siebold WA, Carlson CA, Giovannoni SJ. (2002) SAR11 clade dominates ocean surface bacterioplankton communities. *Nature* **420**: 806-810.

Morris RM, Rappe MS, Urbach E, Connon SA, Giovannoni SJ. (2004) Prevalence of the Chloroflexi-related SAR202 bacterioplankton cluster throughout the mesopelagic zone and deep ocean. *Appl Environ Microbiol* **70**: 2836-2842.

Oksanen J, Blanchet FG, Kindt R, Legendre P, Minchin PR, O'Hara RB *et al.* (2013) vegan: Community Ecology Package. http://CRAN.R-project.org/package=vegan

Pernthaler A, Pernthaler J, Amann R. (2002) Fluorescence In Situ Hybridization and Catalyzed Reporter Deposition for the Identification of Marine Bacteria. *Appl Environ Microbiol* **68**: 3094-3101.

Pinheiro JC, Bates DM. (2002) *Mixed-effects models in S and S-PLUS*, corr. 3. print. (edn.) New York, NY: Springer.

Schattenhofer M, Fuchs BM, Amann R, Zubkov MV, Tarran GA, Pernthaler J. (2009) Latitudinal distribution of prokaryotic picoplankton populations in the Atlantic Ocean. *Environ Microbiol* **11**: 2078-2093.

Tuomisto H. (2010). A diversity of *beta* diversities: straightening up a concept gone awry. Part 1. Defining *beta* diversity as a function of *alpha* and gamma diversity. *Ecography* **33:** 2-22.

Venables WN, Ripley BD. (2002) *Modern Applied Statistics with S.*, Fourth Edition (edn.) New York: Springer.

Wilkinson L. (2011) venneuler: Venn and Euler Diagrams. R package version 1.1-0.
